# Construction and validation of a predictive model for the serum phosphorus reduction after total parathyroidectomy in patients with secondary hyperparathyroidism

**DOI:** 10.3389/fendo.2025.1584602

**Published:** 2025-05-29

**Authors:** Yingnan Feng, Yinghui Zhou, Xiaodong Feng, Qila Sa, Ningyuan Zhang, Wantao Xie, Bailiang Liu, Fengyang Chen, Guangming Cheng, Wei Zhang

**Affiliations:** ^1^ Department of Hepatobiliary Surgery, General Hospital of Northern Theater Command, Shenyang, China; ^2^ Postgraduate College, Dalian Medical University, Dalian, China; ^3^ Postgraduate College, China Medical University, Shenyang, China

**Keywords:** secondary hyperparathyroidism, total parathyroidectomy, hyperphosphatemia, risk factors, predictive model

## Abstract

**Objective:**

We aimed to construct a predictive scoring model for the factors influencing serum phosphorus reduction following total parathyroidectomy (tPTX) in secondary hyperparathyroidism (SHPT) and provide a reference for identifying patients who can successfully correct hyperphosphatemia before surgery.

**Methods:**

The clinical data of 529 patients with SHPT who underwent tPTX were retrospectively analyzed according to the inclusion and exclusion criteria. Univariate and multivariate analyses were conducted to determine the independent factors and establish a predictive scoring model. The receiver operating characteristic curve (ROC) was applied to verify the model in the training and validation groups, respectively.

**Results:**

In the whole group, 315 patients had a significant decrease in serum phosphorus after tPTX. Univariate and multivariate analysis showed that preoperative alkaline phosphatase (AKP), intact parathyroid hormone (iPTH) and free triiodothyronine (FT3) were independent influencing factors to promote the decrease of serum phosphorus after tPTX; Serum phosphorus and bone pain were inhibitory factors (all P<0.05). According to the cut-off value, AKP>193.33 U/L, iPTH>1808 pg/mL, FT3>2.825 pg/mL, serum phosphorus>2.285 mmol/L and bone pain were used to establish the predictive scoring model for serum phosphorus decline. The results showed that the success rate of serum phosphorus reduction was 67.55% at 10~14 points and 95.35% at 15~24 points. The area under ROC curves (AUC) for the training and validation group were 0.818 (95% CI=0.775~0.861) and 0.840 (95% CI=0.780~0.901, both P<0.05).

**Conclusion:**

The established prediction score model for serum phosphorus decrease has a good prediction efficiency which is helpful for the early identification. The model provides important clinical guidance for the postoperative management and treatment of SHPT.

## 1 Introduction

Secondary hyperparathyroidism (SHPT) is one of the common complications in patients with chronic kidney disease (CKD) (1). In these patients, the renal function is impaired, disrupting the balance of calcium absorption and phosphorus excretion in the body. This manifests as persistent hyperphosphatemia, hypercalcemia or hypocalcemia, thereby stimulating the parathyroid gland to secrete parathyroid hormone (PTH). It causes bone destruction and calcium release, which leads to bone pain, skeletal deformities, skin itching and cardiovascular diseases. In severe cases, it will affect the long-term survival of patients (2–4). The current treatment options for SHPT include diet control, drug therapy, dialysis, and surgical treatment.

Drug treatment is mainly classified into phosphate binders, vitamin D and its analogues, and calcimimetics. Phosphate binders can correct hyperphosphatemia to maintain normal serum calcium and PTH levels, and are categorized into calcium-containing (calcium carbonate (5)) and calcium-free (sevelamer (6)) phosphate binders. Vitamin D and its analogues treat SHPT by rectifying the related vitamin D deficiency (7). Commonly used medications include calcitriol, paricalcitol, and alfacalcidol (8, 9). Calcimimetics reduce PTH secretion by activating calcium-sensing receptors, thereby effectively controlling calcium and phosphorus levels (10). Cinacalcet is the most common calcimimetic, which can enhance the sensitivity of calcium-sensing receptors to extracellular calcium and bind allosterically to the receptor to inhibit PTH secretion (11). At present, there are three recognized surgical methods, each with its own pros and cons: total parathyroidectomy (tPTX), subtotal parathyroidectomy (sPTX), and total parathyroidectomy with autotransplantation (tPTX + AT) (12, 13). However, for patients with SHPT who fail to respond to medical therapy, parathyroidectomy (PTX) is often the final choice (14, 15). For dialysis patients who can no longer benefit from kidney transplantation, tPTX is a safe and effective intervention that reduces the recurrence of SHPT and reduces mortality (16). tPTX can be performed also before a scheduled kidney transplantation in order to prevent the development of tertiary HPT and the further impairment of the allograft.

Some patients, on the foundation of long-term SHPT, as the parathyroid glands are stimulated for a long time, the hyperplastic glands have acquired autonomous function and have developed into adenomas or adenoma-like nodules that secrete PTH independently. Even if the kidney transplantation is successful, the parathyroid function cannot return to normal, presenting hypercalcemia, hypophosphatemia, and elevated serum PTH, which is known as tertiary hyperparathyroidism (THPT) (17). tPTX can be performed also before a scheduled kidney transplantation in order to prevent the development of tPTX can be performed also before a scheduled kidney transplantation in order to prevent the development of tertiary HPT and the further impairment of the allograft (18). For such patients, in the clinical setting, it is necessary to pay key attention to the transplant kidney function, renal osteopathy, and the mortality rate of cardiovascular events. Currently, the main treatment approaches are surgical operations and medical treatment mainly based on cinacalcet. The guideline suggests that the surgical approach for THPT treatment should be selected based on the patient’s condition as either tPTx + AT or sPTx (19).

Hyperphosphatemia is a common complication in patients with CKD and a common cause of SHPT. In the general population, the incidence of hyperphosphatemia is about 12% (20), while in patients with end-stage renal disease, it can be as high as 50% to 74% (21). The internal homeostasis of phosphates is mainly maintained through gastrointestinal absorption, bone metabolism, and urine excretion. As CKD progresses, the function of the kidney to excrete phosphorus and to reabsorb calcium declines, leading to the loss of calcium salts and the retention of phosphate salts in the body, which further stimulates the proliferation of parathyroid cells and the secretion of large amounts of PTH, ultimately leading to the development of SHPT. Hyperphosphatemia is capable of inducing the transformation of vascular smooth muscle cells into osteoblasts, promoting calcification of the inner wall of blood vessels, augmenting the risk of coronary artery plaque formation, and resulting in a reduction in the elasticity of peripheral vessels (22, 23). Consequently, it can trigger arteriosclerosis, hypertension, coronary heart disease, left ventricular hypertrophy, and even heart failure (24), and elevate the risk of all-cause mortality and cardiovascular mortality in patients at CKD stage 3 and CKD stage 4 (25). Additionally, hyperphosphatemia can also cause central nervous system reflex hyperactivity, muscle spasms, neuromuscular excitability, even delirium, coma, and tetany symptoms. Research indicates that hyperphosphatemia is an independent risk factor for the progression of chronic kidney disease. Moreover, it can induce renal failure again in patients who have received kidney transplants, imposing a significant economic burden on patients and severely affecting the quality of life of patients with SHPT (26).

Total parathyroidectomy is the most important treatment option for patients with SHPT. At present, the changes of serum calcium in patients with SHPT after tPTX have aroused extensive attention, while insufficient attention has been paid to perioperative serum phosphorus disorders. Previous studies have shown that in some patients, serum phosphorus levels can be significantly improved after PTX treatment, significantly improving their quality of life, but not all patients benefit from it (27). Therefore, it is crucial to identify the factors associated with improved serum phosphorus levels after tPTX treatment. This study retrospectively analyzed the clinical data of patients with CKD secondary to SHPT and hyperphosphatemia before and after surgery, explored the independent factors influencing the decrease of blood phosphorus in patients with SHPT and hyperphosphatemia after surgery, and constructed a comprehensive and efficient preoperative prediction scoring model integrating multiple influencing factors (28). Innovatively, the decrease of blood phosphorus after surgery was stratified in a gradient manner, providing convenience for clinicians’ identification and patients’ understanding. The aim is to provide important references for individualized treatment.

## 2 Data and methods

### 2.1 Research object

Collect the clinical data of 564 SHPT patients who received tPTX treatment at the Northern Theater Command General Hospital of the People’s Liberation Army from April 2015 to April 2024. According to the inclusion and exclusion criteria, 529 cases were selected and randomly divided into training group (n=370) and validation group (n=159) at a ratio of 7:3.

Inclusion criteria (1): Patients with CKD complicated with SHPT (19) (2); The preoperative serum phosphorus level >1.45 mmol/L (4.5 mg/dL) (3); Patients with SHPT who met the surgical indications and received tPTX for the first time (4); iPTH decreased by 80% at 20 minutes after parathyroidectomy (5); All 4 parathyroid glands were removed during operation without ectopic parathyroid glands (6); Patients with complete clinical data.

Excluding criteria (1): Patients with SHPT due to other etiologies (2); Tertiary hyperparathyroidism (3); The preoperative serum phosphorus level ≤1.45 mmol/L (4.5 mg/dL) (4); Primary hyperparathyroidism, parathyroid adenoma or combined malignant tumor (5); Persistent hyperparathyroidism occurred after surgery (6); Postoperative recurrence of hyperparathyroidism was followed by hospitalization and/or reoperation (7); Ectopic parathyroid glands were present or all 4 parathyroid glands were not found during operation (8); Postoperative pathology was not consistent with secondary hyperparathyroidism (9); Patients with incomplete medical records. This study complies with the requirements of the Declaration of Helsinki and has been approved by the Ethics Committee of the General Hospital of the Northern Theater Command of the Chinese People’s Liberation Army. [Approval number: Ethics Y (2024) 127] (Waiver of informed consent has been applied).

Referring to the “Chinese expert consensus on surgical practice of hyperthyroidism in patients with chronic kidney disease (2021 edition)” (19), the indication for surgery in our departmentnm (1): The accompanying clinical symptoms (bone pain, skeletal deformity, muscle weakness, etc.) seriously affect the quality of life (2); Ultrasonography showed at least one enlarged parathyroid gland (volume>500mm^3^ or diameter>1cm) or was clearly localized by MIBI imaging (3); Medical drugs such as cinacalcet, phosphate binders, antiresorptive agents were resistant or ineffective (4); Persistent iPTH>800 ng/mL. Surgical contraindications (1): Patients with severe cardiovascular, respiratory and nervous system dysfunction could not tolerate surgery (2); Severe coagulation dysfunction (3); Acute phase of various infections.

### 2.2 Research parameters

The clinical characteristics of patients such as gender, age, dialysis time, previous renal transplantation, clinical accompanying symptoms (skin itching, bone pain, skeletal deformity (28), muscle weakness, insomnia, restless legs, dry heat), underlying diseases (diabetes, hypertension, heart failure, coronary heart disease) were collected, and the preoperative 25-hydroxyvitamin D, iPTH, Ca, P, Na, K, AKP, albumin, hemoglobin, serum creatinine, thyroid function and echocardiography results. The enrolled patients had been treated with cinacalcet, sevelamer and dialysis before admission, but all patients still had suboptimal serum phosphorus levels. They were no longer taking related medications before and after surgery. A blood test was performed 4 hours after surgery, serum calcium, phosphorus and iPTH were monitored once a day at 5 am within 1 week after operation. The reference range of serum phosphorus was 0.87-1.45 mmol/L. According to the lowest serum phosphorus level after surgery, the patients were divided into postoperative hyperphosphatemia group (>1.45mmol/L) and non-hyperphosphatemia group (≤1.45mmol/L). According to the analysis, the lowest serum phosphorus level after tPTX (*M*=1.27mmol/L) was lower than that before tPTX (*M*=2.33mmol/L). Because the data of blood phosphorus levels both preoperatively and postoperatively presented a skewed distribution, the rank-sum test was adopted for statistical analysis, and we discovered that there was a significant difference between the two data (*P*<0.001). Therefore, we defined the postoperative decrease in serum phosphorus to the normal range as a significant postoperative decrease in serum phosphorus or a successful surgical phosphorus reduction.

Patient information was collected from the hospital’s electronic medical record information system. Clinical concomitant symptoms (pruritus, bone pain, skeletal deformity, muscle weakness, insomnia, restless legs, dry heat) and chronic diseases are all derived from the reports of clinical doctors based on relevant guidelines (binary yes/no). The laboratory analysis indicators collected in this paper are all derived from the data reported by the Laboratory Department of the General Hospital of the Northern Theater Command. The indicators for all patients in this study are uniform. Patients with a history of hypertension, diabetes, and cardiovascular and cerebrovascular diseases were treated under the guidance of relevant departments.

### 2.3 Statistical analysis

**Figure 1:**
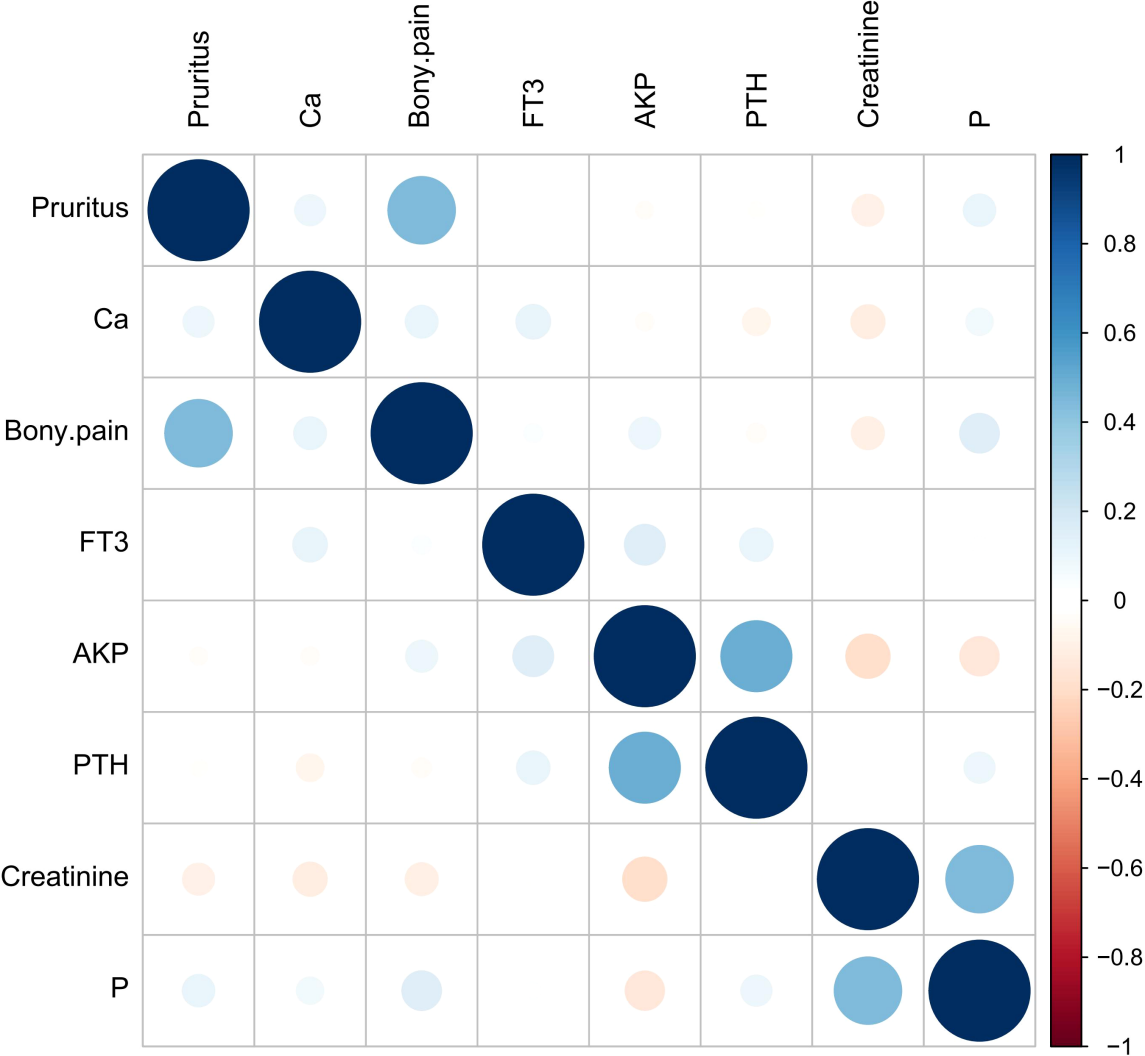
Numerical variable Spearman correlation heat map. Blue represents positive correlation, red represents negative correlation, the size of the circle and the depth of the color represent the strength of the correlation, and the correlation of each numerical variable in the figure is *P*>0.05.

R (version 4.3.3) was used for all statistical analysis. Measurement data were expressed as mean ± standard deviation ( $\bar{x}$ ± *s*) in accordance with the normal distribution, and were expressed as interquartile range [*M*(*IQR*)] in non-normal distribution; Count data were expressed as frequency (percentage). Measurement data conforming to normal distribution were analyzed by *t* test, and non-parametric Kruskal-Wallis (*K*-*W*) test was used for non-normal distribution data; Count data were compared using the χ^2^ test. “Foreign” and “tidyverse” packages were used for data preprocessing, “tableone” package was used to draw the basic characteristics table of clinical data, the “glmnet” package performed univariate Logisitc regression variable screening. Variance inflation factor (VIF) was used to diagnose them for multicollinearity (**Table 1**), and Spearman correlation heatmaps of numerical variables were plotted (**Figure 1**). The selected variables were included in the multivariate Logistic regression to determine the independent influencing factors of postoperative serum phosphorus decline (the significant variables were screened by stepwise method). “Car” and “corrplot” packages were used for collinearity diagnosis and correlation heat maps. On the basis of regression analysis, the “pROC” package was used to draw the receiver operating characteristic curve (ROC) and calculate the area under the curve (AUC) to evaluate the discrimination of the model, and the “optimalCutpoints” package was used to determine the best cut-off value, *P*<0.05 was considered statistically significant. According to the critical value, the data of each influencing factor were classified and included in binary Logistic regression analysis to screen the predictors (**Table 2**). The ***β*** ×4 (rounded) value was used as the score when the predictor was positive, and 0 was used when the predictor was negative (29).Tenfold cross validation was used to evaluate the stability of the model.

**Table 1:** Multiple collinearity diagnosis.

Variable name	Vif
Numerical variables	
iPTH	1.220
Serum calcium	1.054
Serum phosphorus	1.330
Alkaline phosphatase	1.132
Serum creatinine	1.217
FT3	1.061
Categorical variables	
Pruritus	1.257
Bone pain	1.273

All Vif values <5 are considered to have no collinearity between variables, which will not affect the subsequent inclusion in the model.

**Table 2:** Binary Logistic regression analysis was used to analyze the risk predictors of hyperphosphatemia.

Influencing factors	*β*	*OR*	*Z*	*P*	Score
Bone pain	-0.896	0.408	-2.529	0.011	-4
Preoperative iPTH>1 808pg/mL	0.711	2.036	2.432	0.015	3
Preoperative serum calcium>2.285mmol/L	-1.760	0.172	-6.014	<0.001	-7
Preoperative alkaline phosphatase>193.33U/L	1.695	5.446	6.045	<0.001	7
Preoperative FT3>2.825pg/mL	0.869	2.384	2.892	0.004	3
No bone pain/Score≦cutoff					0
Total Score Interval(+11)					-11~13(0~24)

## 3 Results

### 3.1 General information

Of the 529 patients, 315 (59.55%) had a significant decrease in serum phosphorus after surgery, and 214 (40.45%) had no significant decrease in serum phosphorus after surgery. The demographic characteristics, medical history and laboratory indicators of the patients in the training group and the validation group are shown in **Table 3**. Except for the interventricular septal thickness, there were no statistically significant differences in clinical data and serological indicators (all *P*>0.05), which were comparable.

**Table 3:** Comparison of baseline data of SHPT patients in training group and validation group.

Factors	Training group (n=370)	Validation group (n=159)	*t*/*Z*/*χ* ^2^	*P*
Gender (n, %)				
Male	186 (50.3)	78 (49.1)	0.026	0.872
Female	184 (49.7)	81 (50.9)		
Age (years)^a^	48.00 (39.25, 56.00)	47.00 (38.50, 54.00)	-1.422	0.155
Duration of dialysis (years)^a^	8.00 (6.00, 11.00)	8.00 (6.00, 10.00)	-0.142	0.887
Kidney transplantation (n, %)	355 (95.9)	152 (95.6)	<0.001	>0.999
Diabetes mellitus (n, %)	338 (91.4)	151 (95.0)	1.596	0.206
Hypertension (n, %)	61 (16.5)	32 (20.1)	0.781	0.377
Heart failure (n, %)	356 (96.2)	154 (96.9)	0.012	0.914
Coronary Artery Disease (n, %)	350 (94.6)	151 (95.0)	<0.001	>0.999
Clinical symptoms				
Pruritus (n, %)	115 (31.1)	47 (29.6)	0.060	0.806
Bone pain (n, %)	69 (18.6)	33 (20.8)	0.196	0.658
Skeletal deformities (n, %)	342 (92.4)	141 (88.7)	1.529	0.216
Muscle weakness (n, %)	304 (82.2)	137 (86.2)	1.012	0.315
Insomnia (n, %)	328 (88.6)	139 (87.4)	0.065	0.799
Restless leg (n, %)	315 (85.1)	131 (82.4)	0.443	0.506
Thermal Dryness (n, %)	343 (92.7)	148 (93.1)	<0.001	>0.999
Preoperative laboratory test				
iPTH (pg/mL)^a^	1900.00 (1462.25, 1900.00)	1900.00 (1305.00, 1900.00)	-1.737	0.082
Serum calcium (mmol/L)^a^	2.45 (2.31, 2.58)	2.46 (2.32, 2.58)	-0.341	0.733
Serum phosphorus (mmol/L)^a^	2.36 (2.03, 2.78)	2.38 (2.03, 2.78)	-0.538	0.590
Alkaline phosphatase (U/L)^a^	211.11 (139.94, 465.83)	212.60 (128.43, 359.66)	-0.881	0.379
Albumin (g/L)^a^	38.25 (35.73, 40.90)	38.50 (36.00, 40.95)	-1.009	0.313
Hemoglobin (g/L)^a^	108.00 (95.25, 118.00)	109.00 (96.50, 120.00)	-0.330	0.741
Serum sodium (mmol/L)^a^	139.90 (137.83, 141.80)	139.90 (138.40, 141.90)	-0.767	0.443
Serum potassium (mmol/L)^a^	4.63 (4.13, 5.08)	4.68 (4.27, 5.26)	-1.735	0.083
Serum creatinine (mmol/L)^a^	914.89 (761.70, 1150.25)	984.20 (781.30, 1205.02)	-1.576	0.115
25-hydroxyvitamin D (ng/mL)^a^	15.05 (10.53, 21.19)	14.44 (11.24, 21.62)	-0.507	0.612
FT3 (pg/mL)^a^	2.59 (2.28, 2.87)	2.64 (2.34, 2.96)	-1.110	0.267
FT4 (ng/dL)^a^	0.94 (0.81, 1.09)	0.94 (0.83, 1.09)	-0.263	0.793
TSH (mmol/L)^a^	1.56 (1.07, 2.56)	1.43 (1.06, 2.46)	-0.641	0.522
Anti-Tg (IU/mL)^a^	8.26 (5.95, 11.51)	7.72 (5.83, 11.63)	-0.783	0.433
Anti-TPO (IU/mL)^a^	2.26 (1.23, 4.94)	2.75 (1.23, 7.44)	-1.269	0.204
Preoperative ultrasound indicators				
Interventricular septal thickness (mm)^a^	12.00 (11.00, 13.00)	12.00 (11.00, 13.00)	-2.236	0.025
Left ventricular posterior wall thickness (mm)^a^	12.00 (11.00, 12.00)	11.00 (10.00, 12.00)	-1.456	0.145
Left ventricular diameter (mm)^a^	47.00 (43.00, 50.00)	46.00 (43.00, 50.00)	-1.659	0.097

a Median(Q1,Q3). FT3, free triiodothyronine; FT4,free tetraiodothyronine; TSH, thyroid stimulating hormone; Anti-Tg, anti-thyroglobulin antibodies; Anti-TPO, anti-thyroid peroxidase antibody.

### 3.2 Univariate and multivariate analyses

The results of univariate Logistic analysis in the training group showed that pruritus, bone pain, iPTH, serum phosphorus, serum calcium, creatinine, AKP and FT3 could affect the decrease of serum phosphorus after surgery (**Table 4**, all *P*<0.1 to include more possible factors). Multiple collinearity diagnosis showed that VIF values of all variables were <5, and Spearman correlation heat map showed that the correlation between the two variables was *P*>0.05. The statistically significant variables in univariate analysis were included in multivariate Logistic analysis. The results showed that AKP, iPTH and FT3 before operation were independent influencing factors for promoting postoperative serum phosphorus decline, and preoperative serum phosphorus and bone pain were independent influencing factors for inhibiting postoperative serum phosphorus decline (all *P*<0.05, **Table 5**). The cut-off values of AKP, iPTH, FT3 and serum phosphorus calculated by Youden Index were 193.33 U/L, 1–808 pg/mL, 2.825 pg/mL and 2.285 mmol/L respectively.

**Table 4:** Univariate Logistic analysis of influencing factors of serum phosphorus reduction in the training group.

Factors	Non-hyperphosphatemia (n=227)	Hyperphosphatemia (n=143)	*β*	*OR*	*P*
Gender (n, %)					
Male	120 (52.9)	66 (46.2)	0.269	0.764	0.209
Female	107 (47.1)	77 (53.8)			
Age (years)^b^	48.52 (11.48)	47.41 (11.23)	0.009	1.009	0.361
Duration of dialysis (years)^a^	8.00 (6.00, 11.00)	8.00 (5.00, 10.50)	0.045	1.046	0.148
Kidney transplantation (n, %)	219 (96.5)	136 (95.1)	0.343	0.710	0.517
Diabetes mellitus (n, %)	207 (91.2)	131 (91.6)	0.053	1.054	0.889
Hypertension (n, %)	35 (15.4)	26 (18.2)	0.198	1.219	0.486
Heart failure (n, %)	217 (95.6)	139 (97.2)	0.471	1.602	0.434
Coronary Artery Disease (n, %)	214 (94.3)	136 (95.1)	0.166	1.181	0.731
Clinical symptoms					
Pruritus (n, %)	79 (34.8)	36 (25.2)	0.462	0.630	0.052
Bone pain (n, %)	52 (22.9)	17 (11.9)	-0.790	0.454	0.009
Skeletal deformities (n, %)	209 (92.1)	133 (93.0)	0.136	1.146	0.740
Muscle weakness (n, %)	185 (81.5)	119 (83.2)	0.118	1.125	0.674
Insomnia (n, %)	206 (90.7)	122 (85.3)	0.524	0.592	0.111
Restless leg (n, %)	198 (87.2)	117 (81.8)	0.417	0.659	0.156
Thermal Dryness (n, %)	214 (94.3)	129 (90.2)	-0.580	0.560	0.148
Preoperative laboratory test					
iPTH (pg/mL)^a^	1900.00 (1702.00, 1900.00)	1790.00 (1378.50, 1900.00)	0.001	1.001	0.001
Serum calcium (mmol/L)^a^	2.44 (2.30, 2.55)	2.47 (2.34, 2.60)	-0.870	0.419	0.071
Serum phosphorus (mmol/L)^a^	2.18 (1.94, 2.52)	2.64 (2.31, 3.06)	1.748	0.174	<0.001
Alkaline phosphatase (U/L)^a^	294.52 (171.43, 617.60)	149.76 (111.50, 214.68)	0.004	1.004	<0.001
Albumin (g/L)^a^	37.80 (35.35, 40.05)	39.10 (36.25, 42.10)	0.003	1.003	0.532
Hemoglobin (g/L)^a^	108.00 (96.00, 118.00)	108.00 (95.00, 119.00)	0.003	0.997	0.629
Serum sodium (mmol/L)^a^	140.10 (138.00, 141.85)	139.50 (137.55, 141.50)	0.001	1.001	0.873
Serum potassium (mmol/L)^a^	4.51 (4.06, 5.02)	4.74 (4.31, 5.26)	0.014	1.014	0.518
Serum creatinine (mmol/L)^a^	880.30 (712.98, 1090.60)	993.23 (821.92, 1213.45)	0.001	0.999	0.001
25-hydroxyvitamin D (ng/mL)^a^	15.10 (10.70, 21.30)	14.60 (10.50, 21.10)	0.001	0.999	0.370
FT3 (pg/mL)^a^	2.65 (2.34, 3.00)	2.52 (2.22, 2.78)	0.762	2.143	0.001
FT4 (ng/dL)^a^	0.93 (0.81, 1.10)	0.95 (0.81, 1.08)	0.355	1.426	0.223
TSH (mmol/L)^a^	1.58 (1.08, 2.57)	1.56 (1.06, 2.53)	0.011	1.011	0.578
Anti-Tg (IU/mL)^a^	8.52 (6.11, 13.31)	7.85 (5.68, 10.34)	0.002	1.002	0.111
Anti-TPO (IU/mL)^a^	2.31 (1.26, 4.98)	2.09 (1.02, 4.92)	0.005	1.005	0.118
Preoperative ultrasound indicators					
Interventricular septal thickness (mm)^a^	12.00 (11.00, 13.00)	12.00 (11.00, 13.00)	0.037	0.964	0.579
Left ventricular posterior wall thickness (mm)^a^	11.50 (11.00, 12.00)	12.00 (11.00, 12.00)	0.046	0.955	0.553
Left ventricular diameter (mm)^a^	47.00 (43.00, 50.00)	47.00 (44.00, 50.00)	0.008	1.008	0.648

a Median(Q1,Q3).

b Mean ± SD. FT3, free triiodothyronine; FT4, free tetraiodothyronine; TSH, thyroid stimulating hormone; Anti-Tg, anti-thyroglobulin antibodies; Anti-TPO, anti-thyroid peroxidase antibody.

**Table 5:** Multivariate logistic analysis of influencing factors of serum phosphorus reduction.

Influencing factors	*β*	*Z*	*OR* (95%*CI*)	*P*
Pruritus	0.049	0.153	1.050 (0.560~1.976)	0.878
Bone pain	-0.825	-2.134	0.438 (0.201~0.923)	0.033
iPTH	0.001	2.400	1.001 (1.000~1.002)	0.016
Serum calcium	-0.764	-1.319	0.466 (0.147~0.926)	0.187
Serum phosphorus	-1.760	-5.599	0.172 (0.091~0.313)	<0.001
Alkaline phosphatase	0.003	4.097	1.003 (1.002~1.004)	<0.001
Serum creatinine	-0.001	-0.456	1.000 (0.999~1.001)	0.648
FT3	0.832	2.945	2.298 (1.373~4.117)	0.003

### 3.3 Construction and validation of predictive risk models

According to the Logistic regression analysis (**Table 2**), AKP>193.33 U/L, iPTH>1–808 pg/mL, FT3>2.825 pg/mL, serum phosphorus >2.285 mmol/L and bone pain were used as predictors to construct a prediction scoring model for serum phosphorus decline. All patients were scored according to the prediction scoring model. The total score of each patient +11 was taken as a positive number, and the score was divided into 4 scoring intervals according to the quartile of the score. The success rate of serum phosphorus reduction corresponding to each interval was compared and stratified. Final evaluation training group: Scores of 0–6 are in the non-significant group, with a success rate of decline of 21.35%; scores of 7–9 are in the moderate group, with a success rate of decline of 26.47%; scores of 10–14 are in the significant group, with a success rate of decline of 67.55%; scores of 15–24 are in the extremely significant group, with a success rate of decline of 95.35%. The evaluation validation group: Scores of 0–6 are in the non-significant group, with a decline success rate of 12.12%; scores of 7–9 are in the moderate group, with a decline success rate of 25.00%; scores of 10–14 are in the significant group, with a decline success rate of 65.15%; and scores of 15–24 are in the extremely significant group, with a decline success rate of 97.22% (**Table 6**). There were statistically significant differences in the success rate of serum phosphorus reduction among different levels in the training group and the validation group (all *P*<0.05).

**Table 6:** The degree of serum phosphorus reduction was stratified.

Groups	Score interval	Patient (n)	Non-hyperphosphatemia (n)	Accuracy rate (%)	AUC (95%*CI)*
Training group					
non-significant	0~6	89	19	21.35	0.818 (0.775~0.861)
moderate	7~9	44	24	54.55	
significant	10~14	151	102	67.55	
extremely significant	15~24	86	82	95.35	
Validation group					
non-significant	0~6	33	4	12.12	0.840 (0.780~0.901)
moderate	7~9	24	6	25.00	
significant	10~14	66	43	65.15	
extremely significant	15~24	36	35	97.22	

**Figure 2:**
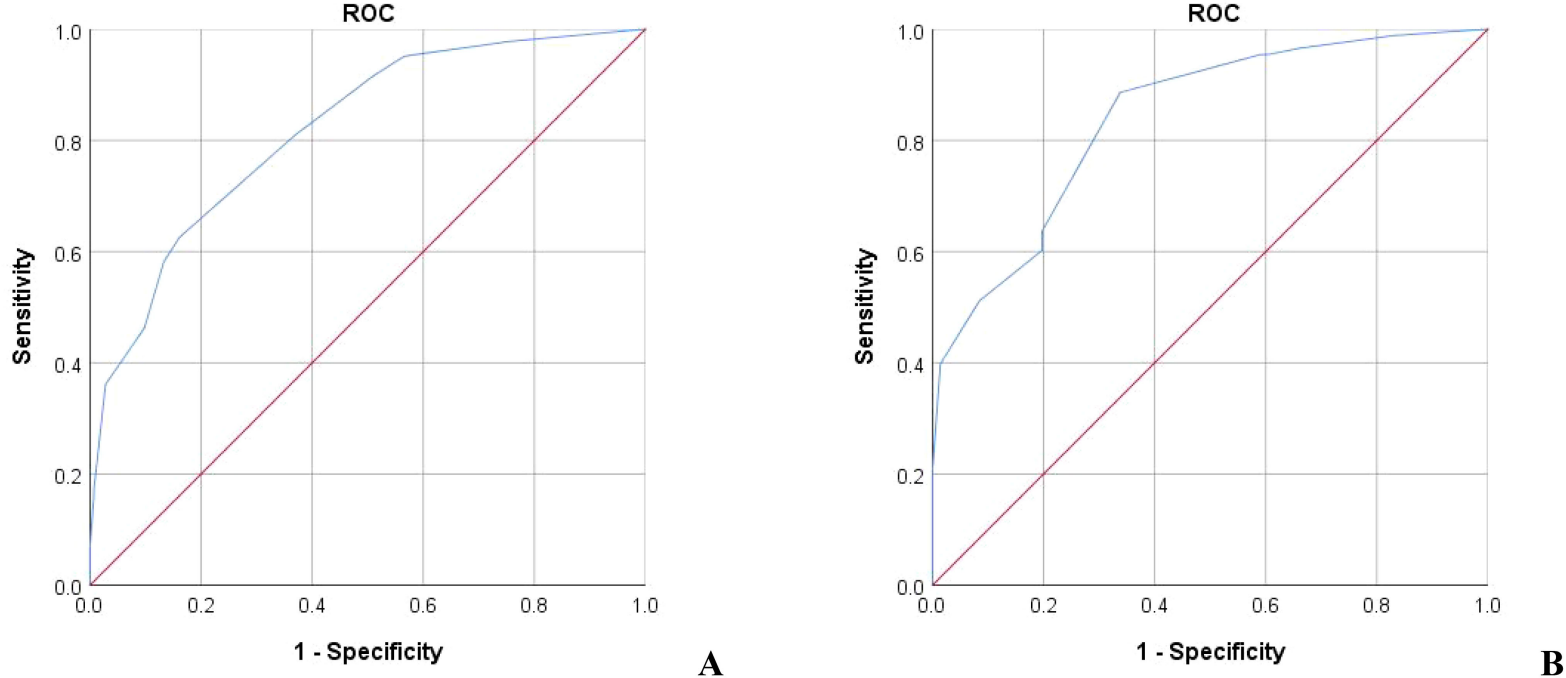
The ROC of the predictive value of the prediction score model for serum phosphorus decline. **(A)** Training group AUC=0.818 **(B)** Validation group AUC=0.840.

It is generally believed that AUC>0.7 can be considered as a good diagnostic effect of the model, and the larger the AUC, the higher the diagnostic accuracy. The ROC curve was drawn with the actual serum phosphorus reduction success result as the state variable and the score value as the test variable. The results showed that the AUC of ROC of the model in the training group was 0.818 (95%*CI* = 0.775-0.861). The AUC of ROC in the validation group was 0.840 (95%*CI* = 0.780-0.901) (**Figure 2**, all *P*<0.05). A positive result indicates that the model predicts correctly, and a negative result indicates that the model predicts incorrectly. Training group: sensitivity was 0.626, specificity was 0.839, accuracy was 0.708; Validation group: the sensitivity was 0.886, the specificity was 0.662, and the accuracy was 0.786.

### 3.4 Ten-fold cross validation

At the same time, tenfold cross validation was used to evaluate the stability of the model. The results showed that the model had excellent diagnostic performance in the ROC curves of the training group and the validation group: the mean AUC of the training set was 0.823; The mean AUC of the validation set was 0.829 (**Table 7**, all *P*<0.05).

**Table 7:** Ten-fold cross validation.

Group	1	2	3	4	5	6	7	8	9	10	Mean
Training	0.824	0.825	0.823	0.821	0.821	0.825	0.827	0.828	0.819	0.818	**0.823**
Validation	0.812	0.799	0.837	0.866	0.846	0.827	0.774	0.791	0.867	0.869	**0.829**

Each value in the table is the AUC value under the ROC curve of each group.

The bolded values represent the average of 10 sets of AUC.

## 4 Discussion

Hyperphosphatemia is a common complication in patients with CKD and secondary hyperparathyroidism in patients with chronic kidney disease. Hyperphosphatemia not only exacerbates hypocalcemia, but also promotes the abnormal deposition of phosphate in renal vasculature, exacerbates renal insufficiency, and is closely associated with inflammation, ectopic vascular calcification, and cardiovascular events (30).

This study analyzed the clinical data of SHPT patients who underwent tPTX in our hospital from April, 2015 to April, 2024. It was found that 59.55% of SHPT patients had significant improvement in blood phosphorus after tPTX. We found that AKP, iPTH, FT3, serum phosphorus and bone pain were predictors of postoperative serum phosphorus improvement in SHPT patients with hyperphosphatemia, while higher preoperative serum phosphorus level and bone pain indicated poor improvement of serum phosphorus after tPTX in SHPT patients.

PTH is the main regulatory hormone of calcium and phosphorus balance, which can increase the blood calcium level and promote the excretion of phosphorus by the kidney. Studies have shown that PTH can promote and stimulate intestinal absorption of calcium and phosphate, release of calcium and phosphate from bone, and promote renal excretion of phosphorus by stimulating bone secretion of fibroblast growth factor 23 (FGF-23) (31, 32). After tPTX, the PTH level of SHPT patients is significantly reduced, which reduces the absorption of phosphate in the intestine and the release of phosphate from the bone, so that hyperphosphatemia can be significantly relieved (33).

As a bone turnover marker, alkaline phosphatase AKP is widely distributed in various organs of human body such as liver, bone, intestine and kidney, reflecting the function of osteoblasts and bone formation in SHPT patients (34), and is an independent factor for vascular calcification (35, 36). Current studies have found that AKP contains a variety of isoforms, among which tissue nonspecific alkaline phosphatase (TNAP) is strongly expressed in bone, liver and kidney, and plays a key role in bone calcification (37). In SHPT patients with little fluctuation of liver function, the increase of AKP indicates that the expression of TNAP is significantly up-regulated. TNAP can degrade extracellular pyrophosphate (PPi) to phosphate, thereby attenuating the inhibitory effect of PPi on vascular calcification. PPi is a major endogenous calcification inhibitor of calcium phosphate crystal formation and growth *in vitro* and *in vivo*, and can significantly inhibit vascular calcification (38). The PPi cycling pathway is: adenosine triphosphate (ATP) is released to the outside of cells through exocytosis and various types of membrane channels. ectonucleotide pyrophosphatase phosphodiesterase (eNPP) hydrolyze ATP to release PPi and adenosine-5 ‘- monophosphate (AMP), PPi is degraded to phosphate via TNAP, and phosphate enters the cell via Sodium-phosphate co-transporter (NaPi) to participate in the synthesis of ATP (39). Renal failure increases the expression of TNAP in vascular smooth muscle and thus accelerates extracellular PPi hydrolysis (40). When extracellular phosphate is insufficient to synthesize sufficient PPi, phosphate from plasma will enter the extracellular fluid to maintain the balance of circulation. In addition, hemodialysis can also remove part of plasma PPi and increase the expression of TNAP (41). Therefore, AKP may exert a phosphorus lowering effect by accelerating PPi hydrolysis.

The higher preoperative iPTH and AKP levels suggest more active bone remodeling and more calcium and phosphorus deposition required for postoperative bone formation. iPTH can reflect bone resorption status in patients with SHPT, and its activation of osteoblasts can activate osteoclasts to promote bone resorption (osteoblast-osteoclast coupling) (42). With the decrease of iPTH of nearly 80% before and after surgery, the coupling activation of osteogenesis and osteoclasts is destroyed, which leads to the cessation of the activity of excess osteoclasts and the increase of the activity of osteoblasts. A large amount of serum calcium phosphorus are absorbed into bone, and then hypocalcemia and hypophosphatemia occur (43). Therefore, the above process may be one of the mechanisms of elevated AKP and iPTH as promoting factors for the decrease of serum phosphorus in SHPT patients after surgery.

Bone pain is a common clinical symptom in patients with SHPT. In uremic patients, the abnormal disorders of osteoclasts and osteoblasts lead to the precipitation of calcium salts and phosphate from bone and the formation of abnormal deposits in extra-bone structures, which leads to the abnormality of serum phosphorus and calcium and causes bone pain (44). Elevated blood phosphorus can stimulate the expression of iPTH gene, promote the activation of osteoclasts, further form a positive closed loop, and aggravate hyperphosphatemia (45). Studies have shown that preoperative bone pain is positively correlated with preoperative iPTH (*r*=0.3), but negatively correlated with postoperative bone alkaline phosphatase (BAKP, *r*=-0.4) and Klotho (*r*=-0.4) (46). Klotho is a protein that can confer tissue specificity to FGF-23 (47). FGF-23 is mainly synthesized by bone cells and osteoblasts and mediates physiological processes by binding to its receptor fibroblast growth factor receptor (FGFR). The involved Klotho affects FGFR sensitivity depending on its concentration. The low BAKP level after surgery indicates poor bone remodeling ability, and the serum phosphorus required for new bone tissue is reduced, while the decreased Klotho level will reduce the phosphorus reducing efficiency of FGF-23 (47). Therefore, preoperative bone pain can lead to higher serum phosphorus level after surgery, which may be one of the mechanisms of bone pain affecting the decrease of serum phosphorus after surgery.

FT3 is generally considered the most active thyroid hormone, and FT3 levels are reduced in up to 75% of patients when renal function declines (48). Some studies have shown that FT3 level is positively correlated with GFR level (49). It is negatively correlated with renal tubular markers and is a protective factor for renal tubular injury (50). Therefore, we inferred that higher FT3 levels represented better renal phosphorus excretion function.

Phosphate, as a signaling molecule, is involved in various cellular processes of bone remodeling, and higher phosphate levels can stimulate continuous secretion of PTH. Phosphate plays a crucial role in the activation and differentiation of osteoblasts. When serum phosphorus and PTH secretion are significantly increased, bone destruction can be stimulated and the activity of osteoclasts and osteoblasts can be activated, both of them are in a state of high speed. After tPTX, the PTH suddenly decreases, and the bone high transport state is improved, accompanied by the decrease of secum phosphorus (33).

One study has suggested that FGF-23 is an independent risk factor affecting the change of secum phosphorus before and after PTX surgery (51). FGF-23 is a novel phosphate-regulating factor secreted by osteoblasts and osteocytes. On the one hand, it promotes urinary phosphorus excretion by down-regulating the expression of sodium and phosphorus transporters in proximal tubular epithelial cells, and on the other hand, it inhibits the synthesis of 1,25(OH)_2_D_3_ by inhibiting the activity of 1α hydroxylase in proximal tubular epithelial cells, thereby reducing intestinal (27). In advanced CKD, the decrease of plasma 1,25(OH)_2_D_3_ reduces Klotho expression in the kidney and parathyroid, leading to the disorder of FGF-23/Klotho axis. The higher the preoperative serum phosphorus level, the lower the Klotho expression, the lower the binding efficiency of FGF-23 to the receptor after PTX, and the poor effect of phosphorus reduction. More studies are needed to confirm the interaction between FGF-23, PTH and 1,25(OH)_2_D_3_ and their effects on bone metabolism and bone phosphorus transport.

The interactions among these indicators such as blood phosphorus, alkaline phosphatase, and PTH are complex and intricate. Although the underlying mechanism cannot be fully elucidated through a single pairwise relationship, it can inspire us to take some actions in clinical practice (1): While routinely testing various indicators of patients after surgery, we suggest that for patients with elevated AKP and PTH before surgery, priority should be given to blood phosphorus testing (2); For patients with obvious bone pain before surgery, bone pain can be scored before and after surgery according to relevant guidelines, or the bone condition can be judged through auxiliary examinations such as DXA and X-rays, and the relationship between the reduction of blood phosphorus and the relief of bone pain over a period of follow-up can be explored (3); For patients with elevated blood phosphorus and FT3 before surgery, regular laboratory tests can be conducted after surgery to determine the long-term effect of the surgery.

In this retrospective clinical study covering 555 SHPT patients, we propose a predictive scoring model based on five preoperative factors that allows prediction of a reduction in phosphorus levels after tPTX based on preoperative clinical and laboratory characteristics. The current issue of the kinetics of serum phosphorus after tPTX has not been fully explored, and the literature tends to focus more on calcium or PTH results after tPTX than on phosphorus, which is novel to us. Compared with previous prediction tools to solve calcium dynamics or hungry bone syndrome, our prediction model has the advantages of simplicity and strong implementation, and is convincing in the number of surgical cases and the accuracy of the prediction results. In addition, we stratified the postoperative serum phosphorus reduction by gradient, which is beneficial to intuitively identify patients with good surgical results, which is also different from other prediction model articles. It is beneficial to clinicians’ judgment and preoperative explanation to patients, and helps some patients to understand the necessity and feasibility of surgery more intuitively. Since the scoring model is clinically simple and stratified by risk intervals, we consider proposing or developing an online calculator, nomogram, or app-based tool to facilitate real-time application by clinicians in the future.

However, this study has several limitations (1): Owing to the constraints of the laboratory, the upper limit of the iPTH measurement values is 1900 pg/mL. In centers with a broader detection range, after incorporating the data of patients with more extreme biochemical characteristics (>1900 pg/mL), the iPTH cutoff value would be higher than the current one. Patients originally within the cutoff value would be excluded as a result of the increase in the cutoff value, and the prediction accuracy of the model would slightly decline. Therefore, the current model accuracy is slightly higher than the actual situation, but would not change its nature as a factor promoting the decrease of serum phosphorus (2); This study is a single-center retrospective study with a single source of samples. Although it has a validation group and ten-fold cross-validation, it still lacks strong external validation (3); This study was a retrospective study and did not include all indicators that may affect serum phosphorus (For instance, perioperative diet, drugs that may influence the changes of blood phosphorus, and blood indicators that are not included in the routine test items, etc.) (4); An unavoidable shortcoming is the loss to follow-up of data from patients with postoperative recurrence of hyperparathyroidism who did not have readmission. There are certain bias and confounding factors in the articles. In the future, multi-center, large-sample external validation and prospective studies are needed to enrich the prediction model, improve the accuracy, and increase the wide applicability of the model.

## 5 Conclusion

In conclusion, this study concluded that preoperative AKP>193.33 U/L, iPTH>1–808 pg/mL and FT3>2.825 pg/mL were independent factors promoting the decrease of serum phosphorus after surgery, while serum phosphorus >2.285 mmol/L and bone pain were independent factors inhibiting the decrease of serum phosphorus after surgery. The established prediction scoring model is simple, convenient and effective, which can provide help for the early identification of serum phosphorus decrease after tPTX in SHPT patients and the early prevention and treatment of patients with high phosphorus after tPTX.

## Data Availability

The original contributions presented in the study are included in the article/supplementary material. Further inquiries can be directed to the corresponding author.

## References

[1] Cunningham J Locatelli F Rodriguez M. Secondary hyperparathyroidism: pathogenesis, disease progression, and therapeutic options. Clin J Am Soc Nephrol. (2011) 6:913–21. doi: 10.2215/cjn.06040710 21454719

[2] Mizobuchi M Ogata H Koiwa F. Secondary hyperparathyroidism: pathogenesis and latest treatment. Ther Apher Dial. (2019) 23:309–18. doi: 10.1111/1744-9987.12772 30411503

[3] Xu Y Evans M Soro M Barany P Carrero JJ. Secondary hyperparathyroidism and adverse health outcomes in adults with chronic kidney disease. Clin Kidney J. (2021) 14:2213–20. doi: 10.1093/ckj/sfab006 PMC8483675 34603697

[4] Morsy MS Dishmon DA Garg N Weber KT. Secondary hyperparathyroidism in heart failure. Am J Med Sci. (2017) 354:335–8. doi: 10.1016/j.amjms.2017.02.008 29078836

[5] Tsukamoto Y Moriya R Nagaba Y Morishita T Izumida I Okubo M. Effect of administering calcium carbonate to treat secondary hyperparathyroidism in nondialyzed patients with chronic renal failure. Am J Kidney Dis. (1995) 25:879–86. doi: 10.1016/0272-6386(95)90570-7 7771484

[6] Lai T Frugoli A Barrows B Salehpour M. Sevelamer carbonate crystal-induced colitis. Case Rep Gastrointest Med. (2020) 2020:4646732. doi: 10.1155/2020/4646732 32774946 PMC7396044

[7] Ojeda López R Esquivias de Motta E Carmona A García Montemayor V Berdud I Martín Malo A. Correction of 25-OH-vitamin D deficiency improves control of secondary hyperparathyroidism and reduces the inflammation in stable haemodialysis patients. Nefrologia (Engl Ed). (2018) 38:41–7. doi: 10.1016/j.nefro.2017.05.008 28673686

[8] Koc H Hoser H Akdag Y Kendir C Ersoy FF. Treatment of secondary hyperparathyroidism with paricalcitol in patients with end-stage renal disease undergoing hemodialysis in Turkey: an observational study. Int Urol Nephrol. (2019) 51:1261–70. doi: 10.1007/s11255-019-02175-5 31161518

[9] Lu CL Shyu JF Wu CC Hung CF Liao MT Liu WC. Association of anabolic effect of calcitriol with osteoclast-derived wnt 10b secretion. Nutrients. (2018) 10:1164. doi: 10.3390/nu10091164 30149605 PMC6164019

[10] Danese MD Lubeck D Belozeroff V Lin TC Desai P Gleeson M. Real world use and effects of calcimimetics in treating mineral and bone disorder in hemodialysis patients. Am J Nephrol. (2020) 51:815–22. doi: 10.1159/000510360 32966995

[11] Bucharles SGE Barreto FC Riella MC. The impact of cinacalcet in the mineral metabolism markers of patients on dialysis with severe secondary hyperparathyroidism. J Bras Nefrol. (2019) 41:336–44. doi: 10.1590/2175-8239-jbn-2018-0219 PMC6788853 31419274

[12] Li C Lv L Wang H Wang X Yu B Xu Y. Total parathyroidectomy versus total parathyroidectomy with autotransplantation for secondary hyperparathyroidism: systematic review and meta-analysis. Ren Fail. (2017) 39:678–87. doi: 10.1080/0886022x.2017.1363779 PMC6446159 28853301

[13] Filho WA van der Plas WY Brescia MDG Nascimento CP Jr. Goldenstein PT Neto LMM. Quality of life after surgery in secondary hyperparathyroidism, comparing subtotal parathyroidectomy with total parathyroidectomy with immediate parathyroid autograft: Prospective randomized trial. Surgery. (2018) 164:978–85. doi: 10.1016/j.surg.2018.06.032 30082137

[14] Yu H Zhang S Hao L Yuan L Wang DG. Safety and short-and long-termefficacy of parathyroidectomy for refractory renal secondaryhyperparathyroidism. Chin J Gen Surgery. (2020) 29:581–8. doi: 10.7659/j.issn.1005-6947.2020.05.009

[15] Lau WL Obi Y Kalantar-Zadeh K. Parathyroidectomy in the management of secondary hyperparathyroidism. Clin J Am Soc Nephrol. (2018) 13:952–61. doi: 10.2215/cjn.10390917 PMC5989682 29523679

[16] Iorga C Iorga CR Andreiana I Bengulescu I Constantin T Strambu V. Advantages of total parathyroidectomy in patients with secondary hyperparathyroidism induced by end stage renal disease. Front Endocrinol (Lausanne). (2023) 14:1191914. doi: 10.3389/fendo.2023.1191914 38075043 PMC10703479

[17] Zhang LX Zhang B Liu XY Wang ZM Qi P Zhang TY. Advances in the treatment of secondary and tertiary hyperparathyroidism. Front Endocrinol (Lausanne). (2022) 13:1059828. doi: 10.3389/fendo.2022.1059828 36561571 PMC9763452

[18] Nakamura M Takiguchi S Uehara S Tomita Y. Outcome of surgical parathyroidectomy for tertiary hyperparathyroidism in kidney transplant recipients: tertiary hyperparathyroidism should not be ignored, for the sake of precious allografts. Ren Fail. (2024) 46:2333919. doi: 10.1080/0886022x.2024.2333919 38575330 PMC10997355

[19] Chinese Thyroid Association, Specialized Committee of Thyroid Disease of Chinese Research Hospital Association. Chinese expert consensus on surgical practice of hyperthyroidism in patients with chronic kidney disease(2021 edition). Chin J ofPractical Surgery. (2021) 41:841–8. doi: 10.19538/j.cjps.issn1005-2208.2021.08.01

[20] Thongprayoon C Cheungpasitporn W Mao MA Sakhuja A Erickson SB. Admission hyperphosphatemia increases the risk of acute kidney injury in hospitalized patients. J Nephrol. (2018) 31:241–7. doi: 10.1007/s40620-017-0442-6 28975589

[21] Leaf DE Wolf M. A physiologic-based approach to the evaluation of a patient with hyperphosphatemia. Am J Kidney Dis. (2013) 61:330–6. doi: 10.1053/j.ajkd.2012.06.026 PMC5505500 22938849

[22] Vervloet M Cozzolino M. Vascular calcification in chronic kidney disease: different bricks in the wall? Kidney Int. (2017) 91:808–17. doi: 10.1016/j.kint.2016.09.024 27914706

[23] Cozzolino M Gallieni M Brancaccio D. Vascular calcification in uremic conditions: new insights into pathogenesis. Semin Nephrol. (2006) 26:33–7. doi: 10.1016/j.semnephrol.2005.06.008 16412823

[24] Ogata H Sugawara H Yamamoto M Ito H. Phosphate and coronary artery disease in patients with chronic kidney disease. J Atheroscler Thromb. (2024) 31:1–14. doi: 10.5551/jat.RV22012 37766573 PMC10776333

[25] Eddington H Hoefield R Sinha S Chrysochou C Lane B Foley RN. Serum phosphate and mortality in patients with chronic kidney disease. Clin J Am Soc Nephrol. (2010) 5:2251–7. doi: 10.2215/cjn.00810110 PMC2994087 20688884

[26] Zhou W Zhang M Ni Z. Acute phosphate nephropathy leading to graft failure. Clin Exp Nephrol. (2019) 23:144–5. doi: 10.1007/s10157-018-1608-9 29951725

[27] Patel A Lee CY Sloan DA Randle RW. Parathyroidectomy for tertiary hyperparathyroidism: A multi-institutional analysis of outcomes. J Surg Res. (2021) 258:430–4. doi: 10.1016/j.jss.2020.08.079 33046234

[28] Guo YY Zhou P Li XL Zhuang DY Yuan J Yue T. Establishment and validation of postoperative risk scoring model for severe hypocalcemia in patients with secondary hyperparathyroidism after surgery. Chin J Gen Surg. (2022) 31:1414–21. doi: 10.7659/j.issn.1005-6947.2022.11.002

[29] Diseases NCRCoK. Summary of China chronic kidney disease mineral and bone disorder guideline. J Nephrol Dialy Transplant. (2019) 28:52–7. doi: 10.3969/j.issn.1006-298X.2019.01.012

[30] Voelkl J Egli-Spichtig D Alesutan I Wagner CA. Inflammation: a putative link between phosphate metabolism and cardiovascular disease. Clin Sci (Lond). (2021) 135:201–27. doi: 10.1042/cs20190895 PMC7796315 33416083

[31] Goltzman D. Physiology of parathyroid hormone. Endocrinol Metab Clinics. (2018) 47:743–58. doi: 10.1016/j.ecl.2018.07.003 30390810

[32] Shimada T Hasegawa H Yamazaki Y Muto T Hino R Takeuchi Y. FGF-23 is a potent regulator of vitamin D metabolism and phosphate homeostasis. J Bone Miner Res. (2004) 19:429–35. doi: 10.1359/jbmr.0301264 15040831

[33] Peters BS Moyses RM Jorgetti V Martini LA. Effects of parathyroidectomy on bone remodeling markers and vitamin D status in patients with chronic kidney disease-mineral and bone disorder. Int Urol Nephrol. (2007) 39:1251–6. doi: 10.1007/s11255-007-9254-2 17680337

[34] Kidney Disease: Improving Global Outcomes (KDIGO) CKD-MBD Update Work Group. KDIGO 2017 clinical practice guideline update for the diagnosis, evaluation, prevention, and treatment of chronic kidney disease-mineral and bone disorder (CKD-MBD). Kidney Int Suppl (2011). (2017) 7:1–59. doi: 10.1016/j.kisu.2017.04.001 30675420 PMC6340919

[35] Shantouf R Kovesdy CP Kim Y Ahmadi N Luna A Luna C. Association of serum alkaline phosphatase with coronary artery calcification in maintenance hemodialysis patients. Clin J Am Soc Nephrol. (2009) 4:1106–14. doi: 10.2215/cjn.06091108 PMC2689878 19423565

[36] Bobryshev YV Orekhov AN SoBenin I Chistiakov DA. Role of bone-type tissue-nonspecific alkaline phosphatase and PHOSPO1 in vascular calcification. Curr Pharm Des. (2014) 20:5821–8. doi: 10.2174/1381612820666140212193011 24533943

[37] Ge YF Yang G Wang NN Zha XM Yu XB Mao HJ. Changes of bone metabolic markers in uremic patients with secondary hyperparathyroidism before andafter parathyroidectomy. Chin J Blood Purif. (2018) 17:588–92. doi: 10.3969/j.issn.1671-4091.2018.09.003

[38] Villa-Bellosta R. Role of the extracellular ATP/pyrophosphate metabolism cycle in vascular calcification. Purinergic Signal. (2023) 19:345–52. doi: 10.1007/s11302-022-09867-1 PMC10247648 35511317

[39] Villa-Bellosta R. Vascular calcification: key roles of phosphate and pyrophosphate. Int J Mol Sci. (2021) 22:13536. doi: 10.3390/ijms222413536 34948333 PMC8708352

[40] Villa-Bellosta R O'Neill WC. Pyrophosphate deficiency in vascular calcification. Kidney Int. (2018) 93:1293–7. doi: 10.1016/j.kint.2017.11.035 29580636

[41] Villa-Bellosta R González-Parra E Egido J. Alkalosis and dialytic clearance of phosphate increases phosphatase activity: A hidden consequence of hemodialysis. PloS One. (2016) 11:e0159858. doi: 10.1371/journal.pone.0159858 27454315 PMC4959680

[42] Goldfarb M Gondek SS Lim SM Farra JC Nose V Lew JI. Postoperative hungry bone syndrome in patients with secondary hyperparathyroidism of renal origin. World J Surg. (2012) 36:1314–9. doi: 10.1007/s00268-012-1560-x 22399154

[43] Wang M Chen B Zou X Wei T Gong R Zhu J. A nomogram to predict hungry bone syndrome after parathyroidectomy in patients with secondary hyperparathyroidism. J Surg Res. (2020) 255:33–41. doi: 10.1016/j.jss.2020.05.036 32540578

[44] Vervloet MG Massy ZA Brandenburg VM Mazzaferro S Cozzolino M Ureña-Torres P. Bone: a new endocrine organ at the heart of chronic kidney disease and mineral and bone disorders. Lancet Diabetes Endocrinol. (2014) 2:427–36. doi: 10.1016/s2213-8587(14)70059-2 24795256

[45] Waheed AA Pedraza F Lenz O Isakova T. Phosphate control in end-stage renal disease: barriers and opportunities. Nephrol Dial Transplant. (2013) 28:2961–8. doi: 10.1093/ndt/gft244 PMC3843343 23901051

[46] Schneider R Steinmetz C Karakas E Bartsch DK Schlosser K. Influence of parathyroidectomy on bone metabolism and bone pain in patients with secondary hyperparathyroidism. Eur Surg Res. (2018) 59:35–47. doi: 10.1159/000486172 29393259

[47] David V Martin A Isakova T Spaulding C Qi L Ramirez V. Inflammation and functional iron deficiency regulate fibroblast growth factor 23 production. Kidney Int. (2016) 89:135–46. doi: 10.1038/ki.2015.290 PMC4854810 26535997

[48] Song SH Kwak IS Lee DW Kang YH Seong EY Park JS. The prevalence of low triiodothyronine according to the stage of chronic kidney disease in subjects with a normal thyroid-stimulating hormone. Nephrol Dial Transplant. (2009) 24:1534–8. doi: 10.1093/ndt/gfn682 19106286

[49] Peters J Roumeliotis S Mertens PR Liakopoulos V. Thyroid hormone status in patients with impaired kidney function. Int Urol Nephrol. (2021) 53:2349–58. doi: 10.1007/s11255-021-02800-2 33682051

[50] Li W Yang Z Li S Jiang S Hu W Wan Z. Free triiodothyronine predicts the risk of developing diabetic kidney disease. BMC Nephrol. (2023) 24:298. doi: 10.1186/s12882-023-03349-1 37821807 PMC10568907

[51] Li SS Mao JP Wang MJ Ding MW Zhang MM Yuan L. Effects of parathyroidectomy on phosphate and bone metabolism in maintenance hemodialysis patients with secondary hyperparathyroidism. Chin J Blood Purif. (2015) 14:256–60. doi: 10.3969/j.issn.1671-4091.2015.05.001

